# Effect of the COVID-19 Lockdown on Children’s Behavior in Makkah, Saudi Arabia

**DOI:** 10.7759/cureus.31234

**Published:** 2022-11-08

**Authors:** Ahdab S Bawashkhah, Afnan A Sulaiman, Maram Alshareef

**Affiliations:** 1 Medicine, Umm Al-Qura University, College of Medicine, Makkah, SAU; 2 Community Medicine and Pilgrims Health, Umm Al-Qura University, College of Medicine, Makkah, SAU

**Keywords:** psychological impact, quarantine, children, mental health, covid-19

## Abstract

Background

Children’s mental health is one of the major concerns during the coronavirus disease 2019 (COVID-19) pandemic. Multiple strategic policies are applied to reduce the spread of the COVID-19 virus, including boundaries closure, social distancing, lockdown, and quarantine. These measures affect the mental health of adults as well as children. In Saudi Arabia, many studies investigated the effect of the COVID-19 pandemic on adults’ mental health, but few were done on children. Children's behavior can be assessed through parents' observation, which can be an important indication of children's mental health.

Objective

This study aimed to assess the psychological impact of the COVID-19 quarantine on children's mental health and to evaluate the effect of familial and social-demographic characteristics on children’s psychology during the COVID-19 crisis in the Makkah region of Saudi Arabia.

Methods and materials

A web-based, cross-sectional voluntary response survey including parents of 576 children aged 15 years and younger. The survey included familial and socio-demographic information as well as a questionnaire examining the behavioral, mental, and emotional changes in children during the COVID-19 quarantine.

Results

Twenty-four point seven percent (24.7%) of children were found to have negative psychological effects due to the COVID-19-associated quarantine in the Makkah region. This prevalence was related to the marital status of the parents, the children's age, and the presence of outdoor space in the house.

Conclusion

This study highlights the importance of psychological support needed for children and their parents during the COVID-19 pandemic. Studies are required to explore whether this psychological impact will subside after the COVID-19 pandemic is over.

## Introduction

In early 2020, the World Health Organization (WHO) classified the new type of coronavirus family, severe acute respiratory syndrome coronavirus 2 (SARS-CoV-2), as a pandemic [1]. Typical COVID-19 patients have respiratory symptoms, including fever, cough, and shortness of breath [2]. COVID-19 is transmitted either directly via droplets and body secretions or indirectly via contaminated surfaces [1,3]. In the Kingdom of Saudi Arabia (KSA), multiple strategic policies have been applied to reduce the spread of the COVID-19 virus, including border closure, social distancing, lockdown, and quarantine [4,5]. These measures, along with other social factors, including the loss of a family member and/or a companion, the increased number of deaths, and the loss of an income source have been shown to have serious effects on the mental health of adults as well as on children [6-8].

Although it is hard for young children to express their feelings and complaints, certain symptoms can be a leading sign to diagnose psychological stress like excessive crying, difficulty in concentrating, and unexplained dizziness. Furthermore, children may experience some behavioral and psychological issues such as attention deficit, social isolation, decreased pro-social behavior, anxiety, boredom, irritability, and fear of COVID-19 [9-11]. A study performed in China during the COVID-19 crisis showed that children from three to six years of age were more likely to worry about the safety of their family members than older groups [12]. However, in all age groups, there were severe psychological symptoms like clinging, lack of attention, and agitation. Also, another study investigated the relationship between decreased outdoor activities and children's mental health following an earthquake and subsequent nuclear reactor accidents [13]. They have shown that decreased outdoor activities are directly correlated with higher psychological illnesses. In fact, during quarantine, outdoor activities were restricted, and children’s interests shifted to electronic games as part of daily entertainment activity [8]. For instance, many studies have shown that increased screen time had an impact on children’s mental and physical health and was associated with an increased prevalence of depression and anxiety [14-18]. In addition, Ali AH et al. showed that electronic games abuse was associated with a drop in the educational level, decreased personal satisfaction, and increased levels of sadness and boredom [14].

In KSA, one study has shown that 44.1% and 27.4% of children/adolescents experienced minimal and COVID-19-related mild post-traumatic stress disorder (PTSD), respectively [19]. This study was conducted during the period of complete lockdown to assess the impact of COVID-19 on children’s social development and mental health in the Makkah region (Saudi Arabia). The study aimed to assess the emotional and behavioral consequences of COVID-19 quarantine on children and the association between familial and socio-demographic characteristics and the psychological condition of children in the Makkah region of Saudi Arabia.

## Materials and methods

The target population included parents who had one or more children between the ages of 3 and 15 years, children who had any guardian other than their parents, who are living in the Makkah region at the time of the study. Parents of children younger than 3 and older than 15 years old and children with chronic diseases or with special needs were excluded from the study. Hence, 576 children met the criteria and were included in the study. The informed consent was distributed by data collectors along with the survey tool. 

Sample size calculation was done using OpenEpi version 3.01, in consideration of the following; the population size, keeping the confidence interval (CI) level at 95%, considering the anticipated % of frequency as 50%, and taking the design effect as 1.

Study design

This was a descriptive cross-sectional study using the snowball sampling technique and was conducted via a web-based electronic survey.

Study tool

The study tool was a modified survey used after permission was obtained from the corresponding author [20]. The survey was distributed in both the Arabic and English languages and was designed to be filled out by the parents. The study included three sections. The first section covered the children’s sociodemographic data and family characteristics. The second section assessed the parental perception of children's psychology during the COVID-19 pandemic. It included a total of 30 questions about changes in the child's behaviors and emotions during quarantine. The responses were taken on the basis of a five-point Likert scale as follows: 1 (never compared to before quarantine), 2 (rarely compared to before quarantine), 3 (sometimes before quarantine), 4 (fairly often compared to before quarantine), and 5 (very often compared to before quarantine). Children with a score of 16 or more were considered psychologically affected by the COVID-19 pandemic. The third section covered the children's screen time by hours, ranging from none to more than 6 hours per day.

The survey was translated into Arabic then back to English through an official translation office and then validated by three expert consultants in community medicine and family medicine. The reliability of the questionnaire’s items was assessed by the test-retest correlation method. The lowest coefficient of stability was 0.68 for the question: My child is anxious, while the highest was 0.82 for the question: My child is afraid to sleep alone.

Data analysis

Statistical analyses were done using IBM SPSS software, version 25 (IBM Corp., Armonk, NY). The descriptive analysis for sociodemographic data was presented as frequencies and percentages of the total population number. The correlation between family characteristics, electronic games use, and the availability of outdoor activities from one side and children’s mental health was assessed using the chi-square test keeping the confidence interval (CI) level at 95%.

## Results

Socio-demographic characteristics of participants

A total of parents submitted information for 576 children who were recruited in the Makkah region, specifically in Makkah and Jeddah cities. The majority of the children were between 7 and 10 years old, whereas most of the parents were between 40 and 49 years old (Table 1). As for the parents, 86.6% were Saudi nationals, 73.8% had access to high education/college, 91.9% were married, and 97.9% shared residence with their kids at the time of the questionnaire.

**Table 1:** Participants' socio-demographic characteristics

(n) %	Children's Characteristic (n) 576
Children's Age Group	
(195) 33.9%	3-6 years old
(227) 39.4%	7-10 years old
(154) 26.7%	11-15 years old
Age Group	
(6) 1.1%	Less than 20 years old
(93) 16.4%	20-29 years old
(182) 32.0%	30-39 years old
(246) 43.3%	40-49 years old
(41) 7.2%	59-69 years old
City	
(466) 80.9%	Makkah
(110) 19.1%	Jeddah
Level of education	
(38) 6.7%	Less than high school
(111) 19.5%	High school or Diploma
(419) 73.8%	Collage or above
Nationality	
(492) 86.6%	Saudi
(72) 13.4%	Non-Saudi
Marital Status	
(522) 91.9%	Married
(26) 4.6%	Divorced
(20) 3.5%	Widow
Occupation	
(48) 8.5%	Healthcare provider
(17) 3.0%	Retired
(142) 25.0%	Fieldwork
(122) 21.5%	Office work
(239) 42.1%	Not working
Does the mother/father live with the child /children?	
(564) 97.9%	Yes
(4) 0.7%	No
(8) 1.4%	Passed away

Socio-familial characteristics of participants

Regarding the prevalence of COVID-19 infection in our population, 19.1% of children had at least one family member infected with COVID-19 (Table 2). Most of the families 52.8% comprised five to seven members and have a monthly income exceeding 10,000 Saudi Arabia Riyals (SAR). Most importantly, 63% of families didn’t have access to outdoor space within their residences. Regarding the use of electronic games, 41.0% of children were allowed three-five hours per day and 35.6% had more than six hours of electronic games use.

**Table 2:** Socio-familial characteristics of participants

(n) %	Presence of a family member diagnosed with COVID-19 (n) 576
Presence of a family member diagnosed with COVID-19	
(110) 19.1%	Yes
(466) 80.9%	No
Number of family members in the house	
(194) 33.7%	2-4
(304) 52.8%	5-7
(67) 11.6%	8-10
(11) 1.9%	More than 10
Family income	
(68) 11.8%	<5000 SAR
(222) 38.5%	5000-10000 SAR
(280) 48.6%	> 10000 SAR
(6) 1.0%	No income
Description of the house	
(129) 22.4%	Outdoor space (garden)
(84) 14.6%	Outdoor space (balcony)
(363) 63%	No outdoor space
House space (number of rooms)	
(11) 1.9%	1-2 rooms
(222) 38.5%	2-4 rooms
(175) 30.4%	5 rooms
(168) 29.2%	More than 5 rooms
My child/children use electronics -- hours per day (n): 432	
(32) 7.4%	1 hour
(52) 12.0%	2 hours
(177) 41.0%	3-5 hours
(154) 35.6%	> 6 hours
(17) 3.9%	None
During the quarantine, the child lived with	
(374) 64.9%	With their parents only
(177) 30.7%	In the big family house
(25) 4.3%	With a family member (uncle or aunt)

Parental perception of children psychology during the COVID-19 quarantine

We investigated the psychological impact of the COVID-19 quarantine on the emotional and behavioral conditions of children and consequently their mental health. Table 3 showed that 24.7% of children were negatively affected by the COVID-19 crisis. For instance, parents have declared that 38.2%, 31.8%, and 34% of children felt “sometimes” more worried, restless, and nervous, respectively. They also noticed that their kids tended to remain silent for a long time (42%). Children were also noted to “fairly often” argue more frequently (23.4%) and to have a tendency to cry easily (16.5%) compared to the pre-COVID-19 situation. Increased boredom and irritability were “fairly often” noticed in 25.9% and 17.9% of children, respectively. Also, parents have confirmed that 19.4% of children were often afraid of contracting COVID-19 infection and 24.1% were becoming more dependent on them on a daily basis (Table 3).

**Table 3:** Parents’ perception of the psychological/behavioral/emotional impact of the COVID-19 quarantine on children

Very Often (n)%	Fairly Often (n)%	Sometimes (n)%	Almost Never (n)%	Never (n)%	Child/Children Behaviors (Total num: 576)
(10) 1.7%	(33) 5.7%	(220) 38.2%	(113) 19.6%	(200) 34.7%	My child is worried
(5) 2.3%	(34) 5.9%	(183) 31.8%	(144) 25.0%	(202) 35.1%	My child is restless
(10) 1.7%	(23) 4.0%	(150) 26.0%	(131) 22.7%	(262) 45.5%	My child is anxious
(6) 1.0%	(5) 0.9%	(89) 15.5%	(154) 26.7%	(322) 55.9%	My child has nightmares
(7) 1.2%	(25) 4.3%	(174) 30.2%	(124) 21.5%	(246) 46.4%	My child is sad
(5) 0.9%	(14) 2.4%	(156) 27.1%	(134) 23.3%	(267) 57.7%	My child is reluctant
(11) 1.9%	(49) 8.5%	(132) 22.9%	(88) 15.3%	(296) 51.4%	My child feels lonely
(7) 1.2%	(20) 3.5%	(114) 19.8%	(131) 22.7%	(304) 52.8%	My child wakes up frequently
(9) 1.6%	(13) 2.3%	(83) 14.4%	(89) 15.5%	(382) 66.3%	My child is very indecisive
(7) 1.2%	(14) 2.4%	(109) 18.9%	(98) 17.0%	(348) 60.4%	My child is uneasy
(15) 2.6%	(64) 11.1%	(196) 34.0%	(127) 22.0%	(174) 30.2%	My child is nervous
(47) 8.2%	(58) 10.1%	(158) 27.4%	(88) 15.3%	(225) 39.1%	My child is afraid to sleep alone
(19) 3.3%	(135) 23.4%	(256) 44.4%	(81) 14.1%	(85) 14.8%	My child argues with the rest of the family
(29) 5.0%	(60) 10.4%	(242) 42.0%	(122) 21.2%	(315) 21.4%	My child is very quiet
(22) 3.8%	(95) 16.5%	(240) 41.7%	(113) 19.6%	(106) 18.4%	My child cries easily
(15) 2.6%	(44) 7.6%	(208) 36.1%	(127) 22.0%	(182) 31.6%	My child is angry
(8) 1.4%	(7) 1.2%	(126) 21.9%	(158) 27.4%	(277) 48.1%	My child asks about death
(7) 1.2%	(16) 2.8%	(105) 18.2%	(136) 23.6%	(312) 54.2%	My child feels frustrated
(20) 3.5%	(149) 25,9%	(239) 41.5%	(72) 12.5%	(96) 16.7%	My child is bored
(19) 3.3%	(103) 17.9%	(240) 41.7%	(108) 18.8%	(106) 18.4%	My child is irritable
(8) 1.4%	(11) 1.9%	(103) 17.9%	(126) 21.9%	(328) 56.9%	My child has sleeping difficulties
(9) 1.6%	(40) 6.9%	(120) 20.8%	(124) 21.5%	(283) 49.1%	My child has no appetite
(8) 1.4%	(26) 4.5%	(159) 27.6%	(139) 24.1%	(244) 42.4%	My child is easily alarmed
(5) 0.9%	(46) 8.0%	(153) 26.6%	(123) 21.4%	(249) 43.2%	My child has difficulty concentrating
(26) 4.5%	(112) 19.4%	(209) 36.3%	(93) 16.1%	(136) 23.6%	My child is afraid of COVID-19 infection
(53) 9.2%	(139) 24.1%	(232) 40.3%	(84) 14.6%	(68) 11.8%	My child is very dependent on us
(4) 0.7%	(15) 2.6%	(112) 19.4%	(104) 18.1%	(341) 59.2%	My child has physical complaints (headache, stomach ache ...)
(9) 1.6%	(18) 3.1%	(85) 14.8%	(103) 17.9%	(361) 62.7%	My child has behavioral problems
(7) 1.2%	(27) 4.7%	(172) 29.9%	(135) 23.4%	(235) 40.8%	My child eats a lot
(15) 2.6%	(36) 6.3%	(143) 24.82%	(113) 19.6%	(269) 46.7%	My child worries when one of us leaves the house
Psychological Impact Among Children During COVID-19					
(434) 75.3%					Negative
(142) 24.7%					Positive

Assessment of the psychological impact of socio-demographic and familial characteristics on children during the COVID-19 crisis

Since results showed that 24.7% were psychologically negatively affected by the COVID-19 quarantine, we investigated more deeply the socio-demographic and familial factors that could contribute to altering children’s mental health during the COVID-19 quarantine. We have found a significant correlation (p=0.01) between the marital status of the parents and the psychological condition of children during the COVID-19 quarantine (Table 4), as children with married parents (67.42%) experienced no or mild COVID-19-related traumatic effect. Moreover, we found a correlation between the presence of outdoor space in the house and kids’ mental health (p=0.04). We found that 45.49% of kids had no outdoor area but experienced no quarantine-related psychological impact (Table 5). However, no association was found between kids’ mental health and the presence of a COVID-19-positive case in the family, nor with house space, family income, and the use of electronic games.

**Table 4:** The association between the socio-demographic features of the participants and the presence of a negative psychological impact

p-value	Socio-demographic characteristics																					Negative psychological impact
0.05	Children age group (years, n = 576)																					
	Total				11-15							7-10				3-6						
				%			n					%		n		%			n			
	142			4.69%			27					10.76%		62		9.20%			53			Yes
	434			22.06%			127					28.64%		165		24.65%			142			No
0.08	Parent's age group (years, n = 568)																					
	Total		50-59				40-49					30-39				20-29			< 20			
		%				n	%			n		%		n		%		n	%	n		
	142	0.70%				4	11.27%				64	7.39%		42		5.29%		30	0.35%	2		Yes
	426	6.52%				37	32.04%				182	24.65%		140		11.09%		63	0.70%	4		No
0.16	Parent's level of education (n = 568)																					
	Total		College or above									High school/ Diploma							Int. school			
			%				n					%				n			%		n	
	142		19.89%				113					4.05%				23			1.06%		6	Yes
	426		53.87%				306					15.50%				88			5.63%		32	No
0.01	Parent's marital status (n = 568)																					
	Total		Widow									Divorced							Married			
			%				n					%		n					%		n	
	142		0.18%				1					0.35%		2					24.47%		139	Yes
	426		3.35%				19					4.23%		24					67.42%		383	No
0.28	Parent's occupation (n = 568)																					
	Total	Not working						Office worker				Fieldworker				Retired			Healthcare worker			
		%				n		%	n			%	n		%		n		%		n	
	142	11.97%				68		4.40%	25			5.47%	31		0.53%		3		2.64%		15	Yes
	426	30.10%				171		17.08%	97			19.55%	111		2.46%		14		5.80%		33	No
0.26	The mother/father lives with the child (n=576)																					
	Total		Passed away									No							Yes			
			%				n					%				n			%		n	
	142		0.00%				0.00					0.17%				1			24.48%		141	Yes
	434		1.38%				8					0.53%				3			73.44%		423	No

**Table 5:** The association between the socio-familial features of the participants and the presence of a negative psychological impact

P-value	Socio-Familial Characteristics, n = 576														Negative Psychological impact
Presence of a family member diagnosed with COVID-19															
0.34	Total	No					Yes								
	n	%			n		%				n				
	142	19.27%			111		5.38%				31				Yes
	434	61.63%			355		13.72%				79				No
Number of family members in the house															
0.25	Total	>10				8-10			5-7				2-4		
	n	%		n		%	n		%		n		%	n	
	142	0.0%		0.0		10.6%	15		53.5%		76		35.9%	51	Yes
	434	2.5%		11		12.1%	52		52.5%		228		32.9%	143	No
Family monthly income (SAR)															
0.87	Total	No income				>10000			5000-10000				<5000		
	n	%		n		%	n		%		n		%	n	
	142	0.7%		1		47.2%	67		38.7%		55		13.4%	19	Yes
	434	1.2%		5		49.1%	213		38.5%		167		11.3%	49	No
Description of the house															
0.04	Total			No outdoor space			Outdoor space (balcony)				Outdoor space (garden)				
	n			%		n	%		n		%		n		
	142			71.1%		101	9.2%		13		19.7%		28		Yes
	434			60.4%		262	16.4%		71		23.3%		101		No
House space (number of rooms)															
0.13	Total	>5		5			3-4				1-2				
	n	%	n	%		n	%		n		%		n		
	142	26.8%	38	24.6%		35	46.5%		66		2.1%		3		Yes
	434	30.1%	130	32.2%		140	35.9%		156		1.8%		8		No
Use electronic games (hours per day)															
0.12	Total			None			>6		3-5		2		1		
	n			%		n	%	n	%	n	%	n	%	n	
	105			2.8%		2	43.4%	46	34.9%	37	8.5%	9	10.4%	11	Yes
	326			4.3%		14	33.1%	108	42.9%	140	13.2%	43	6.4%	21	No
	During the quarantine, the child lived with														
0.17	Total			Other family member (uncle/aunt)			In the big family house				His parents only				
	n			%		n	%		n		%		n		
	142			2.8%		4	26.1%		37		71.1%		101		Yes
	434			4.8%		21	32.3%		140		62.9%		273		No

## Discussion

In this study, the participants were chosen from the Makkah region since it has recorded the highest numbers of COVID-19-positive cases in KSA, as well as the longest complete lockdown period. Herein, the psychological consequences of the COVID-19 crisis on kids’ mental health were assessed. Out of 576 children, 24.7% were found to be significantly affected at the psychological level, 9.2% were between three and six years old, 10.76 % were between seven and 10 years, and 4.69% were in the 11-15 years age group. Nowadays, with easy access to social media and public news, children were regularly updated on the latest COVID-19 death cases, breakouts, and related medical research, which certainly led to increased nervousness and anxiety [17]. A Chinese study aimed to investigate anxiety and depression levels in 399 college and primary school students during the COVID-19 crisis and showed that 34.8% of children experienced mild to moderate nervousness symptoms [18]. Another cross-sectional study among 8079 Chinese students aged 12-18 years during the COVID-19 epidemic period demonstrated that the prevalence of depressive symptoms, anxiety symptoms, and a combination of depressive and anxiety symptoms was 43.7%, 37.4%, and 31.3%, respectively [19].

Further, the emotional and behavioral effects of the COVID-19 quarantine were assessed in children, and it was found that the most common behavioral effects (sometimes, fairly often, and very often) were 71.30% for becoming more dependent on their parents, 70.8% for feeling bored, 71.10% for arguing more frequently with their families, 62.9% for feeling more irritable, and 62% for crying easily. Likewise, a previous study investigating the emotional impact of the quarantine on children and adolescents from Italy and Spain, showed that the most frequent symptoms were difficulty concentrating (76.6%), boredom (52%), irritability (39%), restlessness (38.8%), and nervousness 38% [16].

In order to better understand the contributing factors to COVID-19-associated depression, an analysis was performed to assess the association between the familial and socio-demographic characteristics of the participants from one side, and the negative psychological symptoms observed in children from the other side. Interestingly, kids with married parents exhibit significantly fewer COVID-19-related traumatic effects, which is logical as the loss of one parent would generally result in altered mental health [20]. On the other hand, it is well known that decreased level of outdoor activities was associated with higher psychological illnesses in children and adolescents [21,22]. Surprisingly, the children with limited or no access to outdoor areas showed better mental health. This is probably due to the feeling of safety while staying indoors and the feelings of anxiety and panic that can be associated with outings due to the fear of contracting a COVID-19 infection.

An association between the presence of a positive COVID-19 family member and kids’ psychological status was assessed. It is obvious that the presence of a COVID-19-positive diagnosis in the family will lead to higher stress and nervousness levels because of the fear of contracting the infection. Subsequently, family members will tend to increase the control infection level by excessively augmenting the disinfection and sterilization processes, thereby adding up a new level of stress and anxiety. For instance, previous studies show that having a family member or friend infected with coronavirus was significantly associated with increases in anxiety levels [17]. However, this was not the case in this study and further investigation is needed to examine whether disease severity and duration and the need for hospitalization could affect children’s emotional and behavioral conditions.

In the present study, there was no significant association between electronic games use and psychological condition. However, it is generally well known that long screen time for children results in increased laziness and dispersion as well as decreased emotional stability and concentration. This discrepancy could be explained by the fact that electronic games usage during the COVID-19 pandemic might have a slightly entertaining effect on children suffering from boredom and nervousness due to the lockdown, curfew precautions, and online schooling.

Although children are at low risk to contract COVID-19, they are the most susceptible to suffering from a psychosocial impact during the pandemic. Disturbances in children's lifestyles such as the closure of schools, decreased outdoor activities, insufficient dietary, and improper sleeping habits can induce impatience, distress, annoyance, and various neuropsychiatric symptoms [23]. Therefore, many studies suggested that family therapy after the COVID-19 era is essential for the well-being of the parent's and children's mental health [24]. This includes family engagement in physical activities, adopting new hobbies, or accessing new resources [25].

The limitations of this study include (i) the kids’ gender, which was not included in the survey, (ii) the disproportion in occupations, as the majority of the parents are not working, and (iii) the sample size should be larger, which could allow more elaboration on specific characteristics.

## Conclusions

This present study assessed the psychological impact of the COVID-19 crisis on children in Makkah. The COVID-19 pandemic has been shown to negatively affect the psychological well-being of children, the most vulnerable family component. Therefore, strategies should be taken to empower parents and children during the pandemic. Collaboration between governmental institutions, private sectors, and healthcare professionals is needed to empower parents and children during pandemics by providing psychological support. Currently, after the crisis has ended, reassessment of the mental health of children is needed to encourage early intervention through rehabilitation programs that will help restore the family's well-being.
